# Segregated water observed in a putative fish embryo cryopreservative

**DOI:** 10.1098/rsos.150655

**Published:** 2016-03-02

**Authors:** O. Kirichek, A. K. Soper, B. Dzyuba, W. V. Holt

**Affiliations:** 1 STFC Rutherford Appleton Laboratory, ISIS Facility, Harwell Oxford, Didcot, Oxon OX11 0QX, UK; 2 Faculty of Fisheries and Protection of Waters, University of South Bohemia in Ceske Budejovice, South Bohemian Research Center of Aquaculture and Hydrocenoses, Zatisi 728/II, Vodnany 38925, Czech Republic; 3 Academic Unit of Reproductive and Developmental Medicine, University of Sheffield, Tree Root Walk, Sheffield S10 2SF, UK; 4 Institute of Zoology, Zoological Society of London, Regent’s Park, London NW1 4RY, UK

**Keywords:** common carp embryo, neutron scattering, cryopreservative, water structure

## Abstract

Development of new cryopreservation strategies has major potential in medicine and agriculture and is critical to the conservation of endangered species that currently cannot be preserved. A critical property of any potential cryopreservative solution is its ability to prevent cell-damaging ice formation during cooling and subsequent heating. This study focuses on the freezing behaviour of promising model cryoprotective solutions. We perform neutron scattering analysis, combined with computer modelling, of the water structure after quench cooling these solutions. It is found that water in this solution forms nano-clusters encapsulated by the surrounding matrix of cryoprotectant solute molecules. We posit that these small volumes inhibit ice formation, because water does not have space for the structural relaxation required to crystallize on the timescale of the cooling process.

## 1. Introduction

Under low-temperature conditions (below about 173 K), many plant and animal cells can be preserved successfully for many years. The success of this process, known as ‘cryopreservation’ [1,2], depends on solving the fundamental problem of freezing biological objects, namely, to avoid the formation of lethal intracellular ice. One practical solution to this problem is to disperse cells in aqueous solutions containing penetrating and non-penetrating ‘cryoprotectants’ that inhibit or modify ice formation [3,4]. However, several important types of biological samples have to date resisted attempts to preserve them at low temperature, a case in point being fish embryos [5]. Unlike their mammalian counterparts, which can be routinely frozen and stored in large-scale biobanks, the preservation of fish embryos represents one of the unsolved problems in cryobiology. The storage of female genetic material from important species, races and individuals is currently impossible. The large size and two-compartment nature, with a high yolk content, of fish embryos, coupled with their tough complex membrane systems which give low permeability to cryoprotectants (as an example of a review, see [6]), effectively excludes the penetration of traditional cryoprotectants, and so the embryo is destroyed by ice formation and salt concentration when cooled to low temperature. Hence the problem of finding a satisfactory method of cryopreserving such biological tissue will require a solution that both can be cooled without crystallization and will at the same time penetrate to the inside of the cell with negligible toxic effects.

Vitrification, a quench-cooling process which involves the sudden solidification of water-containing objects, may avoid the formation of either internal or external crystalline ice, and so is a promising alternative approach to cell preservation which is sometimes successful where more traditional freezing methods fail [1,2,7]. While vitrification is relatively well understood as a physical process [8], its application in cryobiology is far from straightforward. In particular, vitrification of cryopreserving solutions is poorly understood at the microscopic level in terms of how water structures itself and molecular dynamics. Therefore in this study, we focus on trying to understand the process of vitrification at the molecular level in cryopreservative solutions which might be appropriate for preservation of fish embryos.

The cryoprotectant solutions used here were formulated during a previous study of fish (common carp) embryo cryopreservation [9]. In that work, the possible modification of the chorion of common carp (*Cyprinus carpio*) embryos by alcalase (proteolytic enzyme) treatment to increase the chorion permeability for cryoprotectants was examined. Two minutes incubation with alcalase was selected as the optimal time for the following experiments, which involved stepwise incubation for 9 min in Hanks’-based solutions containing 1,2-propanediol (PD) and methanol (Met) within the concentration ranges of 5.75–23 vol% and 4.25–17 vol%, respectively. The embryos were either frozen as is or incubated for a further 5 min in a Hanks’-based cryoprotective medium with 23 vol% PD, 17 vol% Met and containing either 20–30 vol% dimethylsulfoxide (DMSO) or 37–57 vol% Met. To explore the toxicity of the different cryoprotective solutions, embryos were transferred into incubators filled with fresh hatchery water. After 1 day of incubation, the hatching rate was determined as a ratio of the number of live, moving embryos to the total number of embryos in the incubators. For the most concentrated cryoprotectants, the hatching rate was in the range 7–40%, while for incubations in media with lower cryoprotectant concentrations, hatching rates were in the range 84–95%. In other words, even at the highest concentrations there was a significant survival probability following treatment with the cryoprotectant solutions.

**Figure 1.**
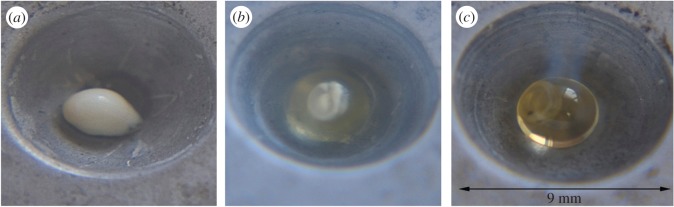
(*a*) Embryo at 77 K dispersed in medium containing 15.41 vol% PD (1,2-propanediol) and 11.39 vol% methanol. Both cryoprotective medium and embryo appear opaque in cooled condition; (*b*) increased concentration of cryoprotectants (23 vol% PD, 17 vol% methanol) leads to appearance of transparent medium and opaque embryo; (*c*) finally, the medium with 23 vol% PD, 17 vol% methanol and 20 vol% DMSO (dimethylsulfoxide) results in transparency of both medium and embryo.

For the work shown here, freezing was achieved using solid surface vitrification, by dropping the embryo onto a metal plate which had been pre-cooled to liquid nitrogen temperature. The use of this series of cryoprotective media with gradually increasing cryoprotectant concentration led to several variants of the sample response on cooling, three of which are shown in figure 1. As shown in figure 1*a*, when the embryo suspended in a medium containing 15.41 vol% PD and 11.39 vol% Met was quench-cooled to liquid nitrogen temperature (77 K), both the cryoprotective media and embryos appeared opaque, signalling either ice formation, protein denaturation, or some other aggregation phenomenon on at least the length scales of visible light wavelengths in both the embryo and the surrounding solution. Increased concentrations of cryoprotectant (23 vol% PD, 17 vol% Met) in the cryoprotective medium led to the appearance of a transparent solution medium but opaque embryo (figure 1*b*). Finally, the use of a cryopreservative medium containing 23 vol% PD, 17 vol% Met and 20 vol% DMSO retained transparency for both the medium and embryo when examined in the cold condition (figure 1*c*). While transparency by itself cannot be regarded as a direct indicator of vitrification over all length scales, the differences between the three cases are quite striking. Protein denaturation, some other aggregation phenomena or ice crystallization will almost certainly lead to a degree of opaqueness [10]. Hence it is clear that whatever physical processes are taking place on quench cooling in these three cases, they are not the same for the different cryopreservatives used. Based on these preliminary results, therefore, these three putative solutions for fish embryo crypreservation represent useful experimental possibilities for the study of solution behaviour when quench-cooled.

Because interpretation of the optical transparency of an object during cooling cannot be used as direct confirmation of the presence or the absence of ice or other heterogeneities, further study is needed. In particular, because microcrystals or other aggregates (of dimension ∼ nm) could potentially be on a scale which is not accessible to optical inspection, and because such heterogeneities might play a decisive role in cryopreservative behaviour, by initiating unstable and damaging ice growth to larger length scales, a specific probe of nano-scale structure is required. Total neutron scattering, with its ability to uncover atomic arrangements at the 0.01–10 nm length scale and to ‘see’ the hydrogen atoms in water and other molecules of the solution, can shed light on the extent of ice crystal formation during cooling, while at the same time giving detailed insight into the way the molecules in these cryoprotectant solutions are organized. This information is vital for understanding the vitrification process in complicated biological objects such as fish embryos, and has the potential to direct the search for better aqueous media for cryopreservation.

A recent study has investigated the way one of these solutions behaves on quench cooling followed by gradual heating [11]. Generally, it was found the vitrified solution undergoes a glass transition on heating around 150 K, as observed in both differential scanning calorimetry and diffraction measurements, with subsequent crystallization to ice on heating through 200 K. The extent and speed of this crystallization depended on the isotopic state of the water and the number of cooling/heating cycles attempted. In this study, we investigate the structure of the quenched cryopreservative solutions at 77 K.

## 2. Material and methods

### 2.1 Sample preparation

We prepared 2 ml of each of the solutions described in table 1. The required materials were obtained from Sigma-Aldrich, used without further purification, and mixed by volume to the required concentrations. (The pure cryopreserving solutions were investigated on their own, none of the samples contained biological material.) Each solution was sealed in a container made of TiZr ‘zero’ alloy—this material is used because it lacks coherent scattering for neutrons and so presents a nearly flat background contribution. It is also highly corrosion resistant. The sample and its container were crash-cooled to 77 K by immersing in liquid nitrogen. The time required to reach thermal equilibrium was less than 40 s, judged by the degree of liquid nitrogen boiling. After keeping the sample in the liquid nitrogen for another 2 min, the container was quickly withdrawn and loaded into the variable temperature insert of a top-loading liquid helium cryostat which was already installed on the neutron beam line. In order to prevent condensation of air and water vapour during the loading of the sample into the cryostat, helium gas flowing through the insert heat-exchanger was temperature controlled at approximately 100 K. Once the sample container was installed the insert was evacuated and a small portion of helium gas was added back in to provide a thermal link between the insert heat-exchanger and the sample container. The temperature of the insert was quickly reduced to 77 K and then controlled at this level during the subsequent neutron scattering measurements. The temperature of the sample cell achieved 77 K within approximately 5 min but another 30 min of sample equilibration was allowed before the neutron diffraction data were collected. The neutron scattering background signal from the empty TiZr container, empty cryostat and empty instrument were subtracted from the experimental data, using separate measurements, and the data put on an absolute scale of differential scattering cross section by comparison with scattering from a slab of vanadium. The neutron scattering data were obtained on the SANDALS diffractometer [12] at the ISIS Facility, STFC Rutherford Appleton Laboratory, UK.

**Table 1.** Compositions and results of quenching at 77 K for the samples used in this study. PD=1,2 propanediol, C_3_O_2_H_7_; MeOH = methanol, CH_3_OH; DMSO = dimethylsulfoxide, (CH_3_)_2_SO. The subscripts show the *volume* fractions of each component. (-h) or (-d) indicates whether that molecule was fully protiated or fully deuteriated.

sample number	reference number	sample composition	result of quenching
1	47159	Pure PD-h	glass (no Bragg peaks)
2	47127	Pure PD-d	glass (no Bragg peaks)
3	47133	(PD-d)_23_+(H_2_O)_77_	crystal (Bragg peaks)
4	47218	(PD-h)_23_+(MeOH-h)_17_+(DMSO-d)_20_+(H_2_O)_40_	glass (no Bragg peaks)
5	47213	(PD-h)_23_+(MeOH-h)_17_+(DMSO-d)_20_+(D_2_O)_40_	glass (no Bragg peaks)
6	47194	(PD-h)_23_+(MeOH-d)_17_+(DMSO-d)_20_+(D_2_O)_40_	glass (no Bragg peaks)
7	47181	(PD-d)_23_+(MeOH-d)_17_+(DMSO-d)_20_+(D_2_O)_40_	glass (no Bragg peaks)
8	47234	(MeOH-h)_17_+(DMSO-d)_20_+(H_2_O)_63_	crystal (Bragg peaks)
9	47225	(MeOH-h)_17_+(DMSO-d)_20_+(D_2_O)_63_	crystal (Bragg peaks)

### 2.2 Data interpretation

Scattering data like these represent a weighted sum over a set of site–site, or *partial*, structure factors, *H*_*αβ*_(*Q*), where *α*, *β* represent the pair of atom sites for each term, and *Q* is the wavevector change in the scattering experiment:

2.1 $$F(Q)=\sum_{\alpha ,\beta <\alpha }(2-\delta_{\alpha \beta })c_{\alpha }c_{\beta }b_{\alpha }b_{\beta }H_{\alpha \beta }(Q),$$

with *c*_*α*_ the atomic fraction and *b*_*α*_ the neutron scattering length for atom type *α*. *H*_*αβ*_(*Q*) is the Fourier transform of the corresponding site–site radial distribution function [13]:

2.2 $$H_{\alpha \beta }(Q)=4\pi \rho \int_{0}^{\infty }r^{2}(g_{\alpha \beta }(r)-1)\frac{\sin Qr}{Qr}\;\text{d}r,$$

where *ρ* is the total atomic number density in the system. In terms of these radial distribution functions, the coordination number of *β* type atoms around an *α* type atom at the origin, *N*_*αβ*_(*r*), is defined by the integral

2.3 $$N_{\alpha \beta }(r)=4\pi \rho c_{\beta }\int_{0}^{r_{\max}}r^{2}g_{\alpha \beta }(r)\;\text{d}r,$$

where *r*_max_ is typically the position of the minimum in *g*_*αβ*_(*r*) beyond the first peak.

For the solutions used in this paper, there are up to 16 distinct atomic sites, giving a total of 136 site–site radial distribution functions to be determined. Interpretation of the data simply by Fourier inverting the total scattering data is, therefore, unlikely to be informative. To overcome this problem, the scattering data from these samples are interpreted in terms of a molecular structure model, using empirical potential structure refinement (EPSR) [14]. This method, which is the analogue of crystal structure refinement, but for disordered materials such as liquids and glasses, builds an atomistic model of the scattering system using standard interaction potentials and models of the individual component molecules, then perturbs those potentials and molecular structures in a manner to bring the simulated radial distribution functions as close as possible to the measured data. It does this while, at the same time, imposing physical constraints on the atomic positions, so as to prevent atomic overlap, and to allow hydrogen-bonding between atomic species where appropriate. Molecular structures are typically derived from a molecular mechanics modelling package, then used, with assumed limits on the inter-atomic overlap, to build and equilibrate the initial simulation box. In the present instance, molecular structure models of methanol, DMSO and water were available from previous work [14,15]. For 1,2-propanediol, there do not appear to have been previous total neutron scattering data on the pure material, so the present data, Samples 1 and 2 in table 1, were used to provide the molecular structure. EPSR simulations were performed on the pure propanediol, Samples 1 and 2, and on the PD/methanol/DMSO/water mixture, samples 4–7. For the former simulations a total of 300 PD molecules were contained in a cubic box of dimension 3.21282 nm, giving an atomic number density of 117.60 atoms nm^−3^. For the latter simulations, a mixture of 193 PD molecules, 260 methanol molecules, 174 DMSO molecules and 1373 water molecules were initially placed at random in a cubic box of dimension 4.9037712 nm giving a number density of 84.196 atoms nm^−3^. For the water molecules, the initial potential parameters were set to those of the TIP4P/2005 potential [16].

## 3. Results and discussion

**Figure 2.**
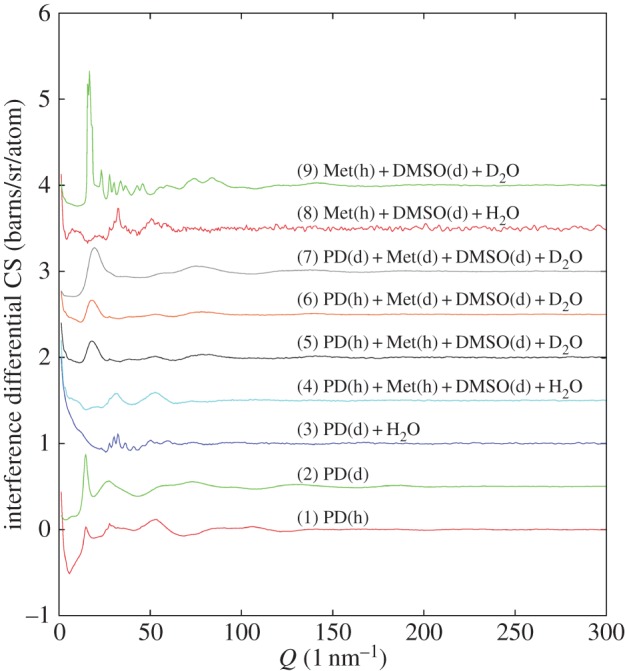
Interference differential cross sections for the cryopreservative aqueous mixtures listed in table 1. Samples 3, 8 and 9 show sharp Bragg peaks, indicating these solutions have crystallized, while the remaining samples, 1, 2, 4–7, show only broad diffuse scattering, which indicates they have retained a disordered, glassy, form. Note how the *Q*-dependence of the data depends sensitively on the isotopic composition of the different samples.

We studied the molecular structure of the quenched aqueous mixtures which had been used in the fish embryo cryopreservation experiments described previously [9]. As is shown in figure 2, only the pure PD (Samples 1 and 2) and the samples with 23 vol% PD, 17 vol% Met and 20 vol% DMSO mixture in water, samples 4–7, produce diffraction patterns typical of a glass structure. The others show sharp peaks indicating some degree of crystallization. This result agrees well with the optical findings shown in figure 1, if it is assumed the opaqueness in these pictures relates to crystalline ice formation. In addition, it is seen that changing between different hydrogen isotopes, but keeping the molecular composition the same, does not affect these general conclusions, indicating the precise isotopic content of the sample does not strongly affect its vitrification or crystallization behaviour.

**Figure 3.**
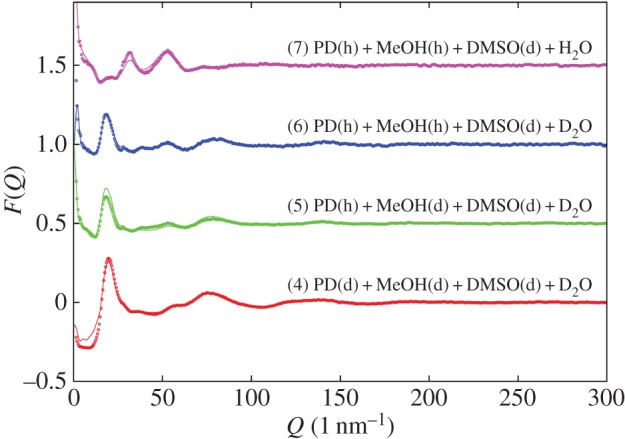
EPSR fits (lines) to the diffraction data (points) for samples 4–7 (23 vol% PD, 17 vol% methanol, 20 vol% DMSO and 40 vol% water), which represent the mixture which has the most promise in this work as a cryopreservative for fish embryos. The data for these graphs are provided in the electronic supplementary material: filename ‘pdmedmwa77k.EPSR.t01’ is the data (points) and ‘pdmedmwa77k.EPSR.u01’ is the EPSR fits (lines).

**Figure 4.**
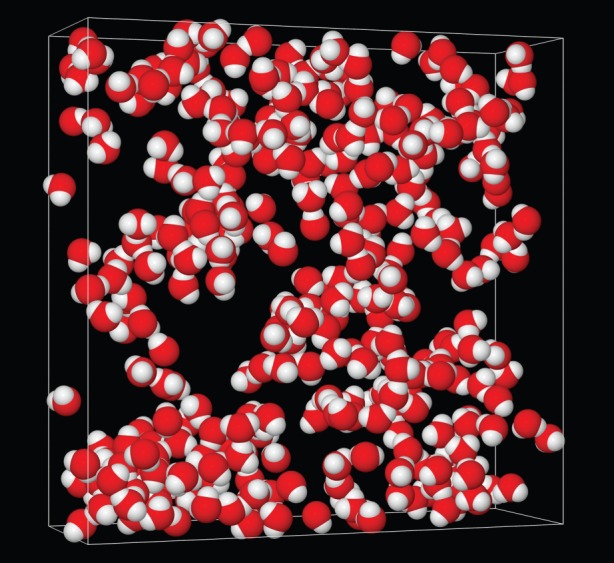
A 1.5 nm slab of the EPSR simulation box for samples 4–7 (23 vol% PD, 17 vol% methanol, 20 vol% DMSO and 40 vol% water), showing only the water molecules. Notice how the water congregates in percolating clusters. It is proposed that it is this inability of the water to form an extended, three-dimensional, network that prevents it from forming ice crystals. In effect, the water becomes ‘confined’ by the surrounding matrix of 1,2-propanediol, methanol and DMSO. The lateral dimensions of the slab are 4.904×4.904 nm.

Figure 3 shows the EPSR fits to the total scattering data that were achieved by this method. Although some minor discrepancies between data and fit remain, the model has clearly captured the main structural features of the data. Hence the model can now be assessed for structural features that might not be visible simply by looking at the scattering data alone. A view of only the water in the simulation box, figure 4, reveals the main outcome of this work, namely that the water, although percolating throughout the system, is actually confined into small volumes of dimensions approximately 1 nm by the surrounding matrix of propanediol, methanol and DMSO. It is posited that it is these small volumes, likely also present in the ambient temperature liquid, and which do not have time to relax and expand during quenching, that prevent the formation of crystalline ice in this system.

In order to characterize the shape of the space occupied by water molecules in this solution, we have performed a so-called ‘void’ analysis on the simulation box [13]. In this method, the simulation box is divided into a large number of volume pixels. In the present case, there were approximately 100 pixels along each of the *x*-, *y*- and *z*-axes, making a total of order 1.0×10^6^ pixels, with the pixel step size set to approximately 0.05 nm. The exact number of pixels used in this routine is optimized depending on the distance criteria being used to define a void. Each pixel is assigned a status of either ‘occupied’ or ‘not occupied’. To be classed as occupied, there must be at least one atom of a specified type within a specified distance from that pixel; otherwise, the pixel is classed as not occupied. In the present case, each water molecule oxygen atom was used to determine if a pixel was occupied or not. At the end, the array of occupied pixels is, to a good approximation, a representation of the space occupied by the water molecules in this solution.

To assess the *shape* of this space an autocorrelation is performed on these occupied pixels, giving a radial distribution function of occupied pixels, otherwise called the ‘volume distribution function’, *g*_*V*_(*r*). Various texts from the theory of small-angle scattering have given functional forms for this correlation function depending on the shape of the space involved (e.g. [17]). The cases of a sphere, a long cylinder and a large flat disc have also been discussed recently [18]. In particular, as discussed in those references, slope of the decay of these correlation functions at *r*=0 gives a measure of the ‘confining length’ of the space represented. For a sphere, the confining length is two-thirds the diameter, while for a long cylinder it is the diameter of the cylinder, and for a large disc it is the thickness of the disc [18]. In the case of a sphere, the correlations disappear for radius values larger than the diameter, but for the long cylinder and flat disc the correlations proceed to much larger distances, in spite of the relatively sharp fall-off at short distances. Hence the detailed shape of the void distribution function is sensitive to the way the pixels that created it are arranged.

**Figure 5.**
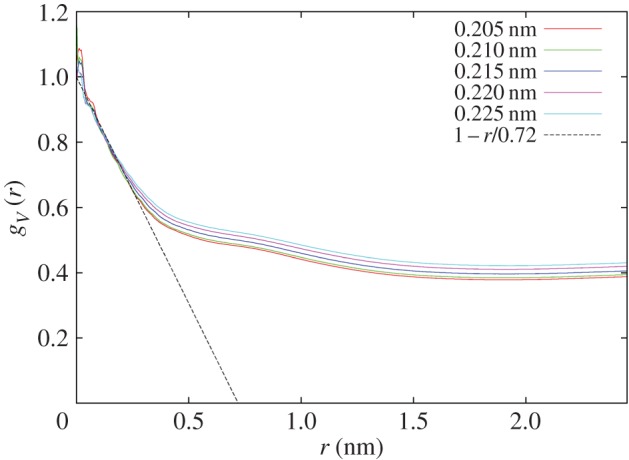
Occupied volume autocorrelation function, *g*_*V*_(*r*), as a function of radius, *r*, for the space occupied by water molecules in a solution of 23 vol% 1,2-propanediol, 17 vol% methanol, 20 vol% DMSO and 40 vol% water for different values of the distance parameter used to determine whether a volume pixel is occupied or not. The distributions have been normalized to the extrapolated value at *r*=0. Also shown is the low *r* asymptote of these distribution functions which indicates a ‘confining length’ of the water to be approximately 0.72 nm. At the same time, long-range correlations to distances on the scale of the simulation box size (4.904 nm) are evident from the large *r* tails to these functions, indicating that although mostly confined to narrow ‘channels’ the water network also percolates throughout the solution. Some intermediate-scale ‘structure’ in these distributions in the region 0.4–1.5 nm is also visible. The small oscillations at very small *r* arise from the finite size of the pixels used to estimate these functions.

A possible limitation here is that the volume distribution function also depends on the distance used to assign whether a pixel is occupied or not. Fortunately, we know from the composition of the sample that the volume fraction of the water is around 40%, hence a natural choice for the defining distance is one which gives approximately 40% occupancy of the volume pixels. In the present work, we used five distances, namely 0.205, 0.210, 0.215, 0.220 and 0.225 nm, which gave fractional occupancies of 0.366, 0.381, 0.396, 0.420, 0.434, respectively, which are all close to the known volume fraction of water. The volume distribution functions obtained in these five cases are shown in figure 5, after normalizing to the estimated value of each function at *r*=0. It can be seen that the initially linear behaviour of these functions at *r*=0 extrapolates to a common value of approximately 0.72±0.02 nm along the *r* axis, suggesting that, since it is only weakly dependent on the choice of radius used to define whether a pixel is occupied or not, it is representative of the lateral extent of the water network. If this is indeed the confining length of the water volume, it corresponds to a water thickness or diameter of approximately two water molecules in any given direction. However, the volume correlations of the water space proceed to much larger values, well beyond the limitation set by the current box, which is *r*=2.452 nm, indicating that although apparently confined to relatively narrow chambers in the solution, the water network is also linked to most other places in the solution by some form of random network, which is exactly what figure 4 attempts to show.

**Figure 6.**
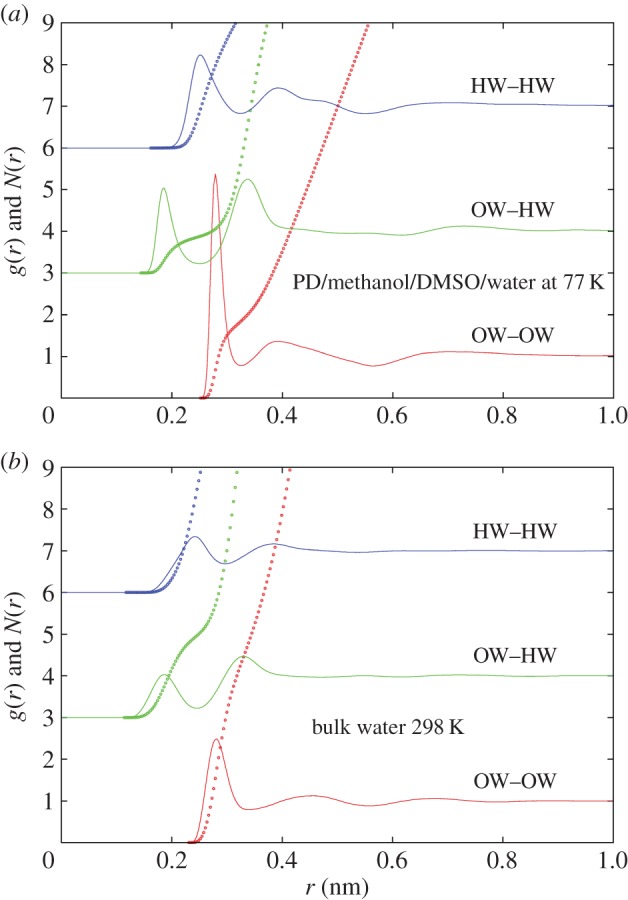
OW–OW, OW–HW and HW–HW (where OW is the water oxygen atom and HW is the water hydrogen atom) radial distribution functions (lines) and running coordination numbers (dots) for 23 vol% PD, 17 vol% methanol, 20 vol% DMSO and 40 vol% water (*a*) compared to the same quantities for bulk water at 298 K (*b*) [19]. The OW–OW and OW–HW coordination numbers quoted in the text are derived using *r*_max_= 0.325 nm and 0.25 nm, respectively. The data for all the *g*(*r*)s in (*a*) are provided in the electronic supplementary material: filenames ‘pdmedmwa77k.EPSR.g01’ and ‘pdmedmwa77k.EPSR.g02’. The data for pure water can be obtained from the cited publication [19].

The structure of the water in this matrix is compared with its structure in the bulk state under ambient conditions [19] in figure 6. Here it is seen that the underlying features of the first neighbour shell in the OW–OW, OW–HW and HW–HW radial distribution functions are in the same positions as in the bulk liquid, but the peaks are markedly sharper in all cases, characteristic of what might be expected in a glass—these features can be compared with those found for the same functions in amorphous ice, [20] and in concentrated sorbitol–water mixtures at low temperature [21]. It is notable however that the second peak in the OW–OW radial distribution function, which is traditionally associated with tetrahedral order in liquid water and low density amorphous ice, has moved to significantly lower *r* values, indicating a likely breakdown in the tetrahedral network induced by the confining matrix. This is a further sign that crystalline ice would be difficult to form in this system unless the rate of cooling had been much slower than used here.

One very clear distinction from the bulk materials is the OW–OW and OW–HW coordination numbers (equation (2.3)) in this system, approximately 1.9 and 0.9, respectively, integrated to 0.325 nm and 0.25 nm respectively. These are factors of 2.2 and 2.1, respectively, lower than in their bulk material counterparts, approximately 4.2 and 1.9, respectively, integrated to the same limits, for ambient bulk water. While this may appear like a large reduction, it has to be borne in mind that in pure water the values of the product *ρc*_*β*_ in equation (2.3) are 0.0333 for *β*=OW and 0.0667 for *β*=HW, whereas in this aqueous mixture the values of *ρc*_*β*_ are 0.0116 for *β*=OW and 0.0233 for *β*=HW, that is, a factor of nearly three times smaller than bulk water. Hence the fact that the actual coordination numbers are only a factor of slightly more than 2 smaller, compared with the bulk liquid, means water in the mixture is coordinating itself by an amount significantly *more*—roughly 30% more—than it would do if this were simply a random mixture of the four components.

## 4. Concluding remarks

The foregoing results provide some general pointers as to how to avoid nucleation of ice when cooling biological organisms, particularly those which are difficult or impossible to cryopreserve, such as fish embryos. However, given that bulk water crystallizes rapidly even when suddenly quenched to liquid nitrogen temperatures, the cooling rate requirement is only part of the requirement. Slowing the rate of crystallization to below what happens in the bulk requires there to be, in addition, an underlying matrix which is able to sufficiently confine the water into small volumes so that ice nucleation becomes impossible on the timescale of the cooling. Concentrated sorbitol–water mixtures apparently provide such a matrix [21], as does the mixture of 1,2-propanediol, methanol, DMSO and water presented here (figure 3). To be successful as a cryoprotectant, however, a third requirement is that the mixture has to be able to penetrate the cell membrane to give sufficient concentration of cryoprotectant inside the cell to prevent ice nucleation *inside* the cell with a minimum of toxicity effects. According to figure 1, this requirement may have been achieved with the 1,2-propanediol, methanol, DMSO and water mixture described here. It achieves cryoprotection because the water is partitioned into connected ‘chambers’ set up by the PD/methanol/DMSO mixture (figure 4). These chambers, joined by a percolating network of water molecules, are too small to allow ice nucleation to occur on the practical timescale for quench cooling in liquid nitrogen that might be achieved in the laboratory. The water structure found in such chambers carries the signature of vitrified bulk water, but there are marked distortions to the hydrogen bond network (figure 6) which prevent the long range formation of crystalline ice.

It must be emphasized that none of the embryos depicted in figure 1, including figure 1*c*, survived heating when attempting to return the embryos to their pre-frozen state. The rate of cooling obviously has to be fast enough to prevent ice nucleation, but there is in addition the likelihood of ice formation on thawing [11]. Hence identifying a suitable cryoprotectant, plus appropriate cooling and heating regimes, for fish embryos is a difficult assignment. The present study sheds some light on why this is so. Our view is that the embryos shown in figure 1 were intact when they went into the vitrification process, and, based on the results of the present study, remained intact in the frozen state in one scenario (figure 1*c*). In that case, the embryo could apparently survive vitrification treatment (i.e. did not show ice formation with vitrification) with the present cryoprotective solution (samples 4–7), and this same solution also does not show ice formation when rapidly cooled to 77 K on its own. Other solutions that showed ice formation in the common carp embryos also showed ice formation when rapidly cooled on their own (samples 3,8,9). However, how we might overcome the remaining ice nucleation obstacles associated with warming remains a project for future study.

## Data accessibility

The data used for this study has been stored in Dryad. http://dx.doi.org/10.5061/dryad.4jv4s.
